# Peritoneal dialysis: update on patient survival

**DOI:** 10.5414/CN108382

**Published:** 2014-10-27

**Authors:** J. Pedro Teixeira, Sara A. Combs, Isaac Teitelbaum

**Affiliations:** Department of Medicine, University of Colorado School of Medicine, Aurora, CO, USA

**Keywords:** hemodialysis, peritoneal dialysis, survival, mortality

## Abstract

Due to ongoing limitations in the availability and timeliness of kidney transplantation, most patients with end-stage renal disease (ESRD) require some form of dialysis during their lifetime. Worldwide, ESRD patients most commonly receive hemodialysis (HD) or one of two forms of peritoneal dialysis (PD), continuous ambulatory PD (CAPD) or automated PD (APD). In this review, we analyze the data available from the last several decades on overall survival associated with HD as compared to PD as well as with CAPD compared to APD. Because of the inherent difficulty in randomly assigning patients to different dialysis modalities, the survival data available are virtually all observational and fraught with many confounding factors and limitations. However, over the last 10 – 15 years as overall survival of dialysis patients has steadily improved and statistical methods to analyze observational data have evolved, a pattern of virtual equivalence in survival among patients on HD vs. PD and on CAPD vs. APD has emerged. As such, impact upon lifestyle and upon quality of life likely should remain the predominant factors in guiding nephrologists and their patients in their choice of dialysis modality.

## Introduction 

While kidney transplantation is the renal replacement therapy of choice when feasible, many patients are not suitable candidates for this procedure. Furthermore, there is a paucity of organs available for transplantation. Thus, most patients with end-stage renal disease (ESRD) will continue to require some form of dialysis – either hemodialysis (HD) or peritoneal dialysis (PD) – as renal replacement therapy. In selecting between these dialysis modalities patients and clinicians may wish to consider survival as one of the factors influencing their decision. In this review we shall discuss the most recent trends in survival for patients using these dialysis modalities. In addition, for patients on PD, we will examine survival on the sub-modalities of continuous ambulatory PD (CAPD) vs. automated PD (APD). 

### Overall survival of ESRD patients on hemodialysis vs. peritoneal dialysis 

There are no randomized controlled trials comparing survival in patients on PD as compared to HD, with one past attempt having proved unsuccessful due to lack of sufficient enrollment [1]. As such, the studies that do exist are observational (Table 1) and, as a result, are inherently confounded by major underlying differences in the two treatment populations. In particular, patients on PD across the world tend to be younger, have fewer co-morbidities, higher hemoglobin concentrations, superior nutritional status, and more residual renal function; they also tend to undergo renal transplantation or modality switch more frequently as compared to patients on HD [2, 3, 4, 5, 6, 7]. Indeed, multiple studies have shown that different conclusions can be drawn from the same patient cohorts depending on the statistical methods used [3, 8, 9, 10]. 

Another complicating factor is that the observational data available have clearly evolved over time as survival in dialysis patients, both PD and HD, has improved over time. The oldest studies, dating from the 1980s, tended to favor HD over the relatively novel PD modality, with studies of patient data from as late as 1989 strongly favoring HD [11]. However, later data from the 1990s showed increasing equivalence [10]. Furthermore, some trends in the 1990s data appeared to emerge. Multiple large registry-based cohort studies from throughout the world, including the US (n = 117,000), Australia and New Zealand (n = 25,000), the Netherlands (n = 16,000), Canada (n = 12,000), and Denmark (n = 6,900), included ESRD patients started on dialysis exclusively or primarily in the 1990s and all showed an initial mortality benefit with PD that lasted for roughly the first 12 – 24 months; after this, point mortality tended to be lower for HD patients, especially those of greater age and co-morbidity [9, 12, 13, 14, 15]. Two prospective but smaller studies, both with 1,000 – 1,250 incident dialysis patients, from similar time periods in the US and The Netherlands had fairly comparable results, namely an equivalent adjusted relative risk of death in the first 1 to 2 years followed thereafter by an increased risk of death in the PD group [6, 16]. Another very large US study of ~ 400,000 patients from the 1990s also reported better outcomes with HD in patients that were older, those with diabetes, and those with other co-morbidities, with better outcomes with PD among younger and “less sick” patients [7], a finding that has subsequently been fairly consistently reproduced elsewhere [4, 6, 9, 12, 13, 14, 15, 16, 17, 18]. 

More recently, three US studies over the last 5 years have used more sophisticated statistics to analyze large retrospective cohorts. The first of these was largely consistent with prior studies, but the last two challenge the understanding that PD tends to outperform HD within the first 2 years, after which point survival is higher with HD. 

In 2010, Weinhandl et al. [18] published a retrospective cohort study of over 6,000 PD patients who were propensity matched with an equal number of HD patients. The PD patients were selected from the nearly 100,000 patients who initiated dialysis in 2003 using the Centers for Medicare and Medicaid Services (CMS) ESRD database. They, however, did not adjust for changes in modality or transplant censoring. The primary outcome, cumulative survival from day 0 of dialysis initiation, was 8% higher in the PD cohort. However, similar to prior studies, the survival benefit decreased over time and was no longer significant after 36 months of follow-up. Unlike some of the prior studies, at no point was HD superior to PD. In addition, the initial benefit of PD was lost in the secondary analysis comparing survival starting at day 90. However, in both the day 0 and day 90 analyses, younger patients and those without diabetes or cardiovascular disease tended to do better with PD in the 1^st^ year whereas older patients and those with diabetes or cardiovascular disease tended to do better with HD in the 2^nd^ and 3^rd^ years. 

**Figure 1.**
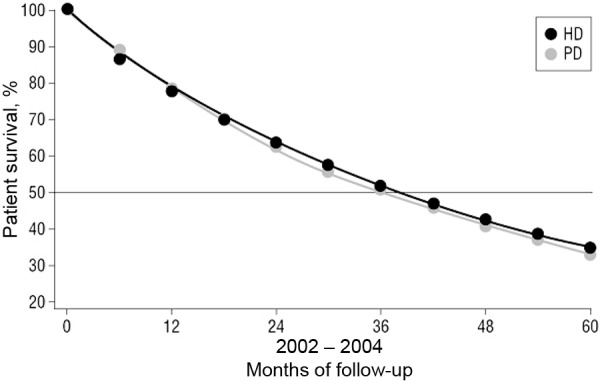
Patient survival by dialysis modality. Adjusted population survival curves comparing the outcome of PD and HD patients from a large US cohort with incident ESRD from 2002 to 2004. From reference [4] with permission. ESRD = end-stage renal disease; HD = hemodialysis; PD = peritoneal dialysis.

In 2011, Mehrotra et al. [4] published the largest contemporary study of its kind, comparing 64,000 PD patients to 620,000 HD patients, all with incident ESRD in the years 1996 – 2004. Comparisons were done separately over three consecutive 3-year time periods (1996 – 1998, 1999 – 2001, and 2002 – 2004) using USRDS data. Analyses were done using a relatively novel statistical method of marginal structural modeling (MSM), first introduced in 2000 [19]. Similar to propensity matching, MSM aims to adjust for selection bias from unmeasured confounders in comparisons made of observation data, but unlike propensity matching it is designed to adjust for confounders that vary over time. Mehrotra et al. [4] used MSM to adjust for censoring from transplant, but importantly the study did not adjust for modality change. As demonstrated in Figure 1, they found in the primary intention-to-treat analysis of the 2002 – 2004 cohort that there was no difference in overall mortality. They did note an improvement in outcomes over time in general in all patients, but this improvement was more pronounced in the PD population. This improvement was seen in all subgroups, but similar to Weinhandl et al. [18] younger, non-diabetic patients with less co-morbidity tended to do better with PD whereas older diabetics with co-morbidities seemed to do better on HD. 

Most recently, Lukowsky et al. in 2013 [3] published a study of over 22,300 HD patients and 1,300 PD patients who started dialysis between July 2001 and June 2004 at US DaVita dialysis centers. They also used the MSM statistical technique to adjust for differential transplantation rates, but, unlike the Mehrotra study above, they also used MSM to adjust for changes to serum hemoglobin and albumin over time and, importantly, for modality changes. In their primary analysis, PD was associated with greater survival, independent of the known confounders including dialysis modality switch and transplant censorship, with a hazard ratio for death of 0.52 at 2 years. Such a large advantage for PD is in stark contrast to the above Mehrotra study, despite both studies being of similar patient populations starting dialysis over similar time periods, both using similar statistical methods, and both being carried out by a similar group of researchers. 

However, the Lukowsky study has some important limitations. First, the study did not report follow-up beyond the initial 2 years. In addition, a few unexplained findings raise questions on the validity of the methods used in the Lukowsky study, particularly in its use of the MSM method to adjust for censorship from modality change. One such notable, unexplained, and unexpected finding was a significant increase in survival noted with modality change, especially when changing from PD to HD, which arguably contradicts clinical reasoning but certainly conflicts with multiple prior studies showing modality change being associated with increased mortality [9, 13, 20]. Also somewhat unexpected and inconsistent with prior reports, the survival advantage in the MSM analysis persisted when stratified by age and diabetes status. Furthermore, when they themselves re-analyzed their DaVita cohort data using conventional (i.e., non-MSM) statistical methods, the survival benefit of PD decreased over time such that there was no difference starting at 2 years. Winkelmayer and Heinze [21] have argued that adjusting for censorship from modality change was likely to enrich the PD cohort with “healthy users” and enrich the HD cohort with “sick stoppers.” In contrast, they argue, the Mehrotra study analyzed the modality-mortality association in a “simpler but more robust” intention-to-treat approach, resulting in the “best estimate to date... of the comparative downstream... survival of a treatment strategy to initiate dialysis on PD vs. one that uses HD”. 

Three separate Canadian studies published in 2011 have suggested a potential explanation for the previously reported early survival advantage of PD over HD during the first 1 – 2 years of therapy. In particular, the advantage of therapy may not be conferred by the modalities themselves but rather results from the complications associated specifically with the use of catheters for vascular access. Indeed, studies had previously clearly documented that use of central venous catheters (CVCs) and, to a lesser degree, of arteriovenous grafts (AVGs), is associated with increased mortality compared to use of arteriovenous fistulae (AVFs) in HD patients [22, 23]. Mendelssohn et al. [24] analyzed 339 incident dialysis patients, of which roughly 25% were started on PD and 75% on HD, and looked at the impact of optimal initiation of dialysis, defined as outpatient initiation via an AVG, an AVF, or a PD catheter, on a composite outcome of death, transfusion, or hospitalization. They found that optimal starts were associated with a greater than 50% decrease in the negative composite outcome over the first 6 months, and, importantly, nearly all (93.7%) of PD patients had optimal starts whereas only a minority (39.5%) of HD patients did. Perl et al. [25] compared ~ 7,400 PD patients, 6,600 HD patients using AVFs or AVGs, and 24,000 HD patients using CVCs, all of whom started dialysis in 2001 – 2008, and found that the HD patients using AVFs or AVGs had a similar or better survival compared to PD patients, whereas the CVC patients had 80% higher mortality than the PD patients. They explicitly conclude that “the use of CVCs in incident HD patients largely accounts for the early survival benefit seen with PD”. Quinn et al. [26] similarly conclude the difference is due to selection bias. Specifically, among a total of over 32,000 patients starting dialysis in Ontario between 1998 and 2006, they were able to identify a minority of ~ 6,500 patients who had at least 4 months of pre-dialysis care and started electively as an outpatient. In this elective cohort there was no difference in overall survival in the HD and PD patients when adjusted for baseline characteristics and the relative risk of death did not change with duration of dialysis. However, the relative risk of death did change over time when they applied the analysis to beyond the elective group. 

Four additional relatively recent studies of dialysis outside the US also, as a whole, support the notion that within modern cohorts long-term survival is equivalent among HD and PD patients. The first of these studies, by Huang et al. in 2008 [17], analyzed over 45,000 and 2,800 patients respectively starting HD and PD in 1995 – 2002 in Taiwan. They found no overall difference long-term survival at 1 – 10 years of follow-up. However, similar to prior studies, they did find increased survival among older and diabetic patients on HD rather than PD in this Taiwanese cohort. Sanabria et al. [27] reported a trend favoring PD but no significant difference in mortality in an adjusted analysis of 900 patients, roughly equally split between HD and PD, who started dialysis in Colombia in 2001 – 2003. Most recently two studies published in 2013, one from Romania and another from Korea, reported on patients who started dialysis in 2008 – 2011. Mircescu et al. [28] in Romania analyzed over 8,200 HD patients and 1,000 PD patients and found no overall survival difference at 36 months and beyond, though survival was higher among the PD patients in the 1^st^ year but higher among the HD patients in years 2 and 3. Choi et al. [2] reported that, when comparing roughly 300 PD patients and 700 HD patients in Korea, the PD patients had improved survival through 2 years of follow-up. Specifically they found a non-significant trend toward decreased mortality using multivariate regression which became statistically significant when using propensity matching; however, they did not have follow-up beyond 2 years in this Korean cohort. 

The finding that PD mortality rates are now equal to that of HD is further supported by the evidence that the improvement in survival over the past two decades among PD patients has significantly outpaced the improvement seen among HD patients. Analyzing USRDS data, Mehrotra et al. [29] reported that among roughly 540,000 incident HD patients and 55,000 incident PD patients, the adjusted rate of death (or transfer to HD) among incident PD patients during the first 12 months of dialysis progressively declined during the period of 1996 – 2003, while the outcomes for HD patients over the same time period remained largely unchanged. Though reasons for this remain unclear, they speculated that it could be due to decreased peritonitis rates, increased dialysis dosage, other quality improvement measures, or more stringent patient selection criteria. Indeed, a decrease in peritonitis rates through various technical advances has been well documented [30, 31, 32], while a general trend of increased PD dose delivered has been seen in the US over the last 2 decades [33]. Unadjusted data from USRDS from as recently as 2002 also show a greater improvement in mortality rates among PD patients than HD patients [34]. Most recently, as noted above, Mehrotra et al. [4], using a similar very large USRDS cohort, reported the secondary finding that survival improved among HD and PD patients between 1996 and 2004, but with a greater increase in the PD cohort. Outside the U.S., Heaf et al. [13] similarly found that, in their Danish cohort of patients starting dialysis between 1990 and 1999, survival improved by 16% in the PD cohort but only by 13% in the HD cohort. 

### Survival of ESRD patients with congestive heart failure: HD vs. PD 

In the past, it was widely believed that patients with ESRD and congestive heart failure (CHF) fared better on PD than HD. This was largely due to several single-center observational studies demonstrating that patients on PD enjoyed lower rates of hospitalization and improved quality of life than those on HD [35, 36, 37, 38]. However, their relative survivals were not assessed until an analysis of USRDS data by Stack et al. in 2003 [39]. This retrospective cohort study included 107,922 incident dialysis patients (at 90 days post initiation) from May 1995 to July 1997. 33% of the patients had CHF (as defined by the CMS medical evidence form) and a total of 93,900 patients were on HD and 14,022 on PD. In both diabetic and non-diabetic patients, compared to HD, PD was associated with significantly higher risk of death in patients with CHF. The effect remained when the data were adjusted for differences in co-morbidity and transplant rates, and were confirmed in both intention-to-treat and as-treated analyses. 

More recently, Sens et al. [40] published data from the French Renal Epidemiology and Information Network (REIN) registry to evaluate survival in HD and PD patients with CHF. The data were prospectively obtained between 2002 and 2008, and patients were followed until the end of 2008. They included incident dialysis patients with CHF as defined by the nephrologist initiating the dialysis and excluded patients with an unplanned first dialysis treatment. A total of 4,401 patients were included in the study, with 3,469 started on HD and 933 on PD. Multivariate survival analysis demonstrated a higher mortality risk with PD than HD among these incident dialysis patients with an adjusted hazard ratio at 90 days of 1.48 (95% CI 1.33 – 1.65). They used propensity scores to attempt to minimize treatment selection bias and incorporated them into the Cox regression analysis as a stratification or adjustment variable. These analyses did not change the results. 

These data seem to suggest that potentially there could be a survival advantage to HD over PD when treating ESRD patients with CHF. However, Mehrotra [41] recently argued that, while there may be an inherent advantage in dialysis modality for this population, it is just as likely that the apparent survival benefit lies in the difference in which the modality is practiced or that there are variables that were not identified or adjusted for in these non-randomized studies. Indeed, treatment of patients with CHF via PD requires careful and frequent adjustments to both the prescription and the patient’s routine, as volume status is likely to be the largest factor affecting these survival differences. 

In conclusion, though the overall mortality of dialysis patients remains high relative to the general population, survival has progressively improved over the past several decades in HD patients and, even more so, in PD patients. Data, including the most contemporary data, seem to suggest that “sicker” patients (i.e., those that are older, diabetic, and have more co-morbidity) may have somewhat better survival on HD whereas “less sick” patients may live longer on PD, though the possibility that these findings stem from residual confounding remains. Importantly, with one notable exception that actually favors PD [3], the sum of the most recent observational data available suggests that overall mortality of HD and PD today is roughly equivalent. This is especially true when one specifically compares PD to the standard of care for HD, provided via a fistula or at least a graft, as the early mortality benefit of PD over HD is likely attributable to catheter-associated morbidity. Of course, given that in many countries only a minority of patients is initiated on HD via a fistula or graft [2, 24, 42], this early mortality benefit of PD should be considered a valid advantage of PD when deciding between PD and HD via a catheter. However, among patients who begin dialysis planning early enough to allow time for a fistula, a graft, or a PD catheter rather than an HD catheter, in the continued absence of randomized trial data, patient preference and impact on lifestyle should remain the most important factors in determining the most appropriate modality of renal replacement therapy. 

### Survival in continuous ambulatory PD (CAPD) vs. automated PD (APD) 

Historically, providers made decisions about PD modality based on patients’ peritoneal membrane characteristics. It was believed that because rapid transporters benefit from multiple short dwell times, APD should be the modality of choice, and conversely, as slower transporters benefit from longer dwells, CAPD should be the chosen modality. However, as patients and providers increasingly opt for convenience, APD has been modified for use in patients with all transport characteristics. Currently, more than 70% of PD patients in the US are treated with APD [43]. 

**Figure 2.**
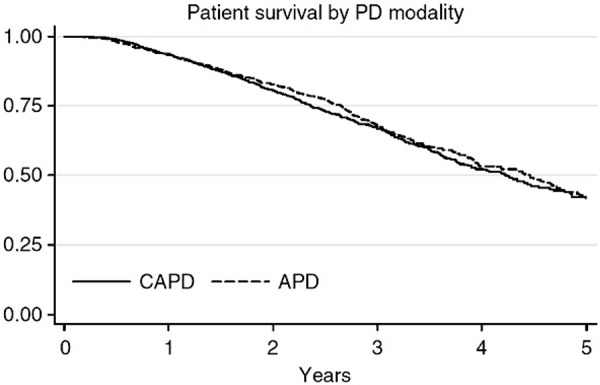
Kaplan-Meier curves of comparative survivals by univariate analysis of incident peritoneal dialysis patients on APD vs. CAPD in New Zealand and Australia. From reference [46] with permission. APD = automatic peritoneal dialysis; CAPD = continuous ambulatory peritoneal dialysis.

There are many studies published that aim to determine if there are survival differences between the two modalities. Similar to the mortality data comparing HD and PD survival, most data comparing survival between CAPD and APD are observational. The only prospective, randomized trial examining mortality outcomes in CAPD vs. APD demonstrated no difference in patient survival [44]. However, the study was underpowered. Most multi-center large-scale observational studies that evaluated mortality differences between CAPD and APD did not demonstrate differences in mortality (Figure 2) [45, 46, 47, 48, 49]. However, the national dialysis registry system in Australia and New Zealand (ANZDATA) also looked specifically at mortality data of PD patients in rapid vs. slow transporters. On multivariate intention-to-treat analysis, they found that there was a lower death risk in rapid transporters treated with APD (adjusted HR 0.56, 95% CI 0.35 – 0.87) and a higher death risk in slow transporters treated with APD (HR 2.19, 95% CI 1.02 – 4.70) [50]. A single-center observational study in Taiwan of 282 incident PD patients found that in patients younger than 65 years of age, those on APD had a significantly lower risk of mortality (HR 0.35, 95% CI 0.16 – 0.75) than those on CAPD [51]. There was no significant difference in mortality between the two modalities in patients 65 years or older. Neither nutritional status nor demographic characteristics could account for difference in the younger patients. In summary, when counseling patients on choosing PD modality, the observational data in general seem to suggest that there is little if any difference in mortality risks between CAPD vs. APD. Additionally, as the majority of patients on PD are intermediate transporters, mortality specific to transporter status is likely to have little effect on everyday practice. 

## Conclusion 

In contemporary cohorts, survival of patients performing HD or PD is similar. Likewise, within PD itself, survival is similar for patients performing CAPD or APD. Therefore, when discussing dialysis modality selection with patients, rather than focusing on length of survival, the focus should be on the quality of survival, i.e., on an analysis of the patient’s lifestyle and the dialysis modality most conducive to it. 

## Acknowledgments 

The authors would like to extend thanks to Angela Keniston, MSPH, for her assistance in statistical interpretation of some of the above studies. 

## Conflict of interest 

The authors declare no conflict of interest. 

**Table 1.** Summary of studies comparing overall survival of PD compared to HD. Restricted to observational studies of incident dialysis patients beginning in 1987 or later, sorted by 1^st^ year of dialysis initiation.

First author, publication year	Location, years RRT initiated	Sample size	Follow-up	Study design	Statistical method	Key outcomes	Comments
Liem et al. 2007 [14]	The Netherlands, 1987 – 2002	n = 16,643 (HD = 10,841; PD = 5,802)	Mean 2.4 ± 2.1 years	Cohort study using national registry	Intention-to-treat analysis using multivariate Cox regression model	Overall adjusted mortality did not differ, but was highly dependent on diabetes status, age, and time since dialysis initiation.	PD was most favorable in youngest non-diabetics during months 3 – 6 of RRT; HD was most favorable in oldest diabetics after 15 months of RRT.
Fenton et al. 1997 [9]	Canada, 1990 – 1994	n = 10,633 (HD = 7,792; PD = 2,841)	Up to 5 years (mean not reported)	Cohort study using national registry	Multivariate Poisson regression	PD had decreased adjusted mortality with the benefit concentrated on the first 2 years of follow-up.	Difference not found when using an intention-to-treat Cox regression model; benefit of PD decreased with age and presence of diabetes; modality switch was associated with an increased risk of death.
Heaf et al. 2002 [13]	Denmark, 1990 – 1999	n = 4,921 (HD = 3,281, PD = 1,640)	Up to 10 years (mean not reported)	Registry-based cohort study	Intention-to-treat analysis using Kaplan-Meier and Cox proportional hazard models	Overall adjusted mortality was lower for PD vs. HD, but difference was confirmed to first 2 years of RRT.	Advantage of PD was lowest in diabetic patients, becoming non-significant; change in modality was associated with increased mortality; overall survival in both groups increased by 14% during study period.
McDonald et al. 2009 [15]	Australia and New Zealand, 1991 – 2005	n = 25,287 (HD = 14,733; PD = 10,554)	Mean 2.7 years	Registry-based cohort study	Cox regression	Overall mortality rates were significantly lower during the 1st year among those treated with PD, but after 12 months PD use was associated with increased mortality.	Younger patients without comorbidities had a mortality advantage with PD treatment.
Collins et al. 1999 [12]	U.S., 1994 – 1996	n = 117,158 (HD = 99,048; PD = 18,110)	Mean of 16.2 and 13.8 months, for HD and PD, respectively	Cohort study using national (Medicare) registry	Adjusted interval Poisson and Cox regressions	PD survival was initially significantly better than HD, though benefit dissipated with time, with PD remaining superior or comparable within the first 2 years.	PD performed best in younger, non-diabetic, females.
Jaar et al. 2005 [16]	U.S. (81 dialysis centers in 19 states), 1995 – 1998	n = 1,041 (HD = 767; PD = 274)	Up to 7 years; mean 2.4 years	National prospective cohort study	Cox proportional hazards regression, stratified by clinic	Risk for death did not differ between PD and HD patients in year 1, but became significantly higher among PD patients in year 2.	Risk of death associated with PD vs. HD use was higher in patients with cardiovascular disease, but not in patients without cardiovascular disease.
Huang et al. 2008 [17]	Taiwan, 1995 – 2002	n = 48,629 (HD = 45,820, PD = 2,809)	Up to 11 years (mean not reported)	Retrospective cohort study using national registry	Intention-to-treat analysis using Cox proportional hazard and Kaplan-Meier models	No significant difference in long-term survival in adjusted analysis.	Diabetic patients and patients older than 55 experienced better survival on HD than on PD.
Mehrotra et al. 2011 [4]	U.S., 1996 – 2004 (divided into three 3-year cohorts)	n = 684,426 (HD = 620,020; PD = 64,406)	Up to 5 years, with median of 24 to 30 months in each cohort	Cohort study using national (USRDS) registry	Intention-to-treat analysis using marginal structural model adjusting for censoring from transplant (but not modality change)	In the 2002 – 2004 cohort, there was no difference in overall mortality.	An improvement in outcomes was found over time in all patients, but the improvement was more pronounced with PD; older diabetics with co-morbidities seemed to do better on HD.
Termorshuizen et al. 2003 [6]	The Netherlands, 1997 – 2002	n = 1,222 (HD = 742; PD = 480)	Outcomes reported up to 48 months (mean not reported)	Multicenter, prospective, observational, cohort study	Multivariate Cox regression	No statistically significant difference in adjusted mortality between HD and PD during the first 2 years; thereafter, RR of death was higher for PD patients relative to HD patients.	This tendency was observed especially among patients 60 years of age or older.
Sanabria et al. 2008 [27]	Colombia, 2001 – 2003	n = 923 (HD = 437, PD = 486)	Range of 2 – 5 years (mean not reported)	Retrospective cohort study	Intention-to-treat analysis using Kaplan-Meier and Coxproportional hazard models	No significant difference in mortality in adjusted analysis.	The survival trend favored PD, despite higher rates of diabetes, lower SES, and more co-morbidity in PD group.
Lukowsky et al. 2013 [3]	U.S., 2001 – 2004	n = 23,718 (HD = 22,360; PD = 1,358)	Defined at 24 months	Cohort study using corporate (DaVita) registry	Marginal structural model, adjusting for modality change, differential transplant rates, and time-varying lab measurements	PD was associated with 48% lower 2-year mortality than HD.	Modality change was associated with significant increase in survival; findings did not hold with conventional (non-MSM) Cox proportional analysis.
Weinhandl et al. 2010 [18]	U.S., 2003	n = 6,337 pairs (HD = PD)	Up to 4 years; mean 2.3 years	Matched-pair retrospective cohort study using CMS registry	Intention-to-treat analysis using propensity matching; did not adjust for censoring from transplant or modality change	Cumulative survival was 8% higher in the PD cohort, though survival benefit decreased over time and was no longer significant after 36 months.	Younger patients and those without diabetes or cardiovascular disease tended to do better with PD in year 1 whereas older patients and those with diabetes or cardiovascular disease tended to do better with HD in years 2 – 3.
Mircescu et al. 2014 [28]	Romania, 2008 – 2011	n = 9,252 (HD = 8,252, PD = 1,000)	Range of 1 – 5 years (mean not reported)	Cohort study using national registry	Intention-to-treat analysis using Kaplan-Meier and Cox proportional hazard models	PD survival was higher among in the first year, HD was survival higher in years 2 – 3, and thereafter survival in both groups was equivalent.	HD survival was higher in younger diabetics (in contrast to subgroup analyses of most other studies).
Choi et al. 2013 [2]	Korea, 2008 – 2011	n = 1,060 (HD = 736; PD = 324)	Mean 16.3 ± 7.9 months	Prospective observational cohort	Intention-to-treat analysis using multivariate Cox regression with a subset (n = 278 pairs) matched by propensity score	PD had trend toward decreased mortality using multivariate regression which was statistically significant when using propensity matching.	The most common cause of death was infection; ~ 60% of HD was via a CVC; no follow-up reported beyond 2 years.

CMS = Centers for Medicare & Medicaid Services; CVC = central venous catheter; HD = hemodialysis; PD = peritoneal dialysis; RR = relative risk; RRT = renal replacement therapy; SES = socio-economic status.
